# Incidence and Characterization of Concealed Cardiac Amyloidosis Among Unselected Elderly Patients Undergoing Post-mortem Examination

**DOI:** 10.3389/fcvm.2021.749523

**Published:** 2021-11-23

**Authors:** Aldostefano Porcari, Rossana Bussani, Marco Merlo, Guerino Giuseppe Varrà, Linda Pagura, Davide Rozze, Gianfranco Sinagra

**Affiliations:** ^1^Cardiovascular Department, Center for Diagnosis and Treatment of Cardiomyopathies, Azienda Sanitaria Universitaria Giuliano-Isontina, University of Trieste, Trieste, Italy; ^2^Cardiothoracic Department, Center for Diagnosis and Treatment of Cardiomyopathies, Institute of Pathological Anatomy and Histology, Azienda Sanitaria Universitaria Giuliano-Isontina, University of Trieste, Trieste, Italy

**Keywords:** transthyretin (ATTR) amyloidosis, light chain (AL) amyloidosis, epidemiology, histology, red flags, amyloidosis, diagnosis

## Abstract

**Background:** The prevalence of cardiac amyloidosis (CA) is unknown.

**Aims and Methods:** We sought to (a) determine the prevalence of CA in unselected patients ≥75 years undergoing autopsy, (b) characterize cardiological profiles of CA and non-CA patients by providing clinical-histological correlations, and (c) compare their cardiological profiles. After dedicated staining, the localization (interstitial or vascular) and the distribution (non-diffuse or diffuse) of amyloid deposition were analyzed. Cardiological data at last evaluation were retrospectively assessed for the presence of CA red-flags.

**Results:** CA (50% light chains, 50% transthyretin) was found in 43% (*n* = 24/56) of the autopsied hearts. Atria were involved in 96% of cases. Amyloid localized both at the perivascular and interstitial levels (95.5 and 85%, respectively) with a slightly predominant non-diffuse distribution (58% of cases). Compared to the other patients, CA patients had a more frequent history of heart failure (HF) (79 vs. 47%, *p* = 0.014), advanced NYHA functional class (III-IV 25 vs. 6%, *p* = 0.047), atrial fibrillation (68 vs. 36%, *p* = 0.019), discrepancy between QRS voltage and left ventricular (LV) thickness (70 vs. 12%, *p* < 0.001), thicker LV walls (15 vs. 11 mm, *p* < 0.001), enlarged left atrium (49 vs. 42 mm, *p* = 0.019) and restrictive filling pattern (56 vs. 19%, *p* = 0.020). The presence of right ventricular amyloidosis seemed to identify hearts with a higher amyloid burden. Among the CA patients, >30% had ≥3 echocardiographic red-flags of disease.

**Conclusion:** CA can be found in 43% of autopsied hearts from patients ≥75 years old, especially in patients with HF, LV hypertrophy and atrial fibrillation.

## Introduction

Cardiac amyloidosis (CA) is an increasingly recognized cause of heart failure (HF) and mortality, resulting from the progressive deposition of misfolded proteins (1, 2), mainly immunoglobulin light chains (AL) and transthyretin (ATTR) (3). Most epidemiological data come from historical post-mortem studies that report a disarming prevalence of concealed CA in 25% of unselected adults >80 years old and in 32% of patients >75 years old with HF with preserved ejection fraction (HFpEF) (4). However, these studies had a mainly histological focus; none of them were conducted on unselected populations, nor did they provide a correlation between histological and clinical data. The epidemiology of the disease is currently unknown, especially regarding ATTR, but recent investigations using cardiac scintigraphy with bone tracers suggest that CA is a relatively frequent condition (5–7).

We therefore designed this study to (a) determine the prevalence of CA in an unselected population ≥75 years old who were autopsied, (b) characterize the cardiological profile of CA patients by providing clinical-histological correlations, and (c) compare the cardiological profiles of patients with and without CA.

## Materials and Methods

### Study Design and Specimen Collection

Data of all patients referred for post-mortem evaluation at the University Hospital of Trieste (Institute of Pathological Anatomy and Histology, Cardio-Thoracic Department, Italy) between April and June 2019 were collected in a prospective electronic database and retrospectively analyzed. Patients <75 years old, deceased because of severe septic conditions or with post-mortem alterations that would have compromised an adequate microscopic evaluation were excluded. According to these criteria, the final population included patients referred for post-mortem examination by the Departments of Internal Medicine, Oncology and Geriatrics. None of the patients were referred or followed-up by the Department of Cardiology. All patients signed an informed consent document to allow for the use of anonymized personal information for research purposes. The study was conducted according to the Declaration of Helsinki ethical guidelines.

### Macroscopic Cross-Sections and Histological Analysis

**Figure 1:**
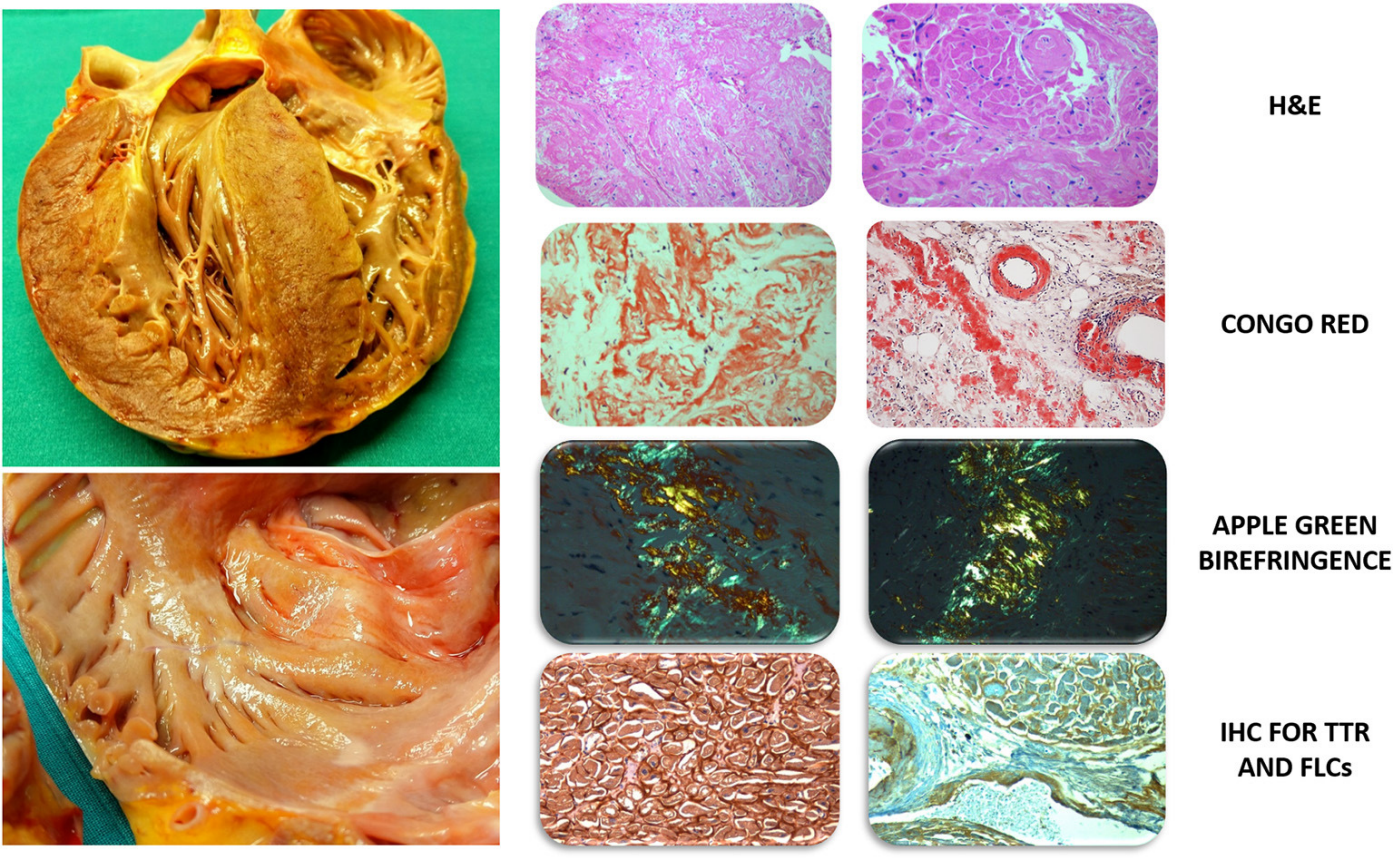
Gross evaluation of unfixed heart and histological characterization with dedicated staining. **Left Top:** massive cardiac hypertrophy of the left ventricle involving the subvalvular aortic region extending to the right ventricle, the papillary muscles and the inter-atrial septum. The heart exhibits a heterogeneous color with pale areas and faint yellowish tone. **Bottom:** increased atrial wall thickness with diffuse granulations on the endocardial surface and nodular deposits of amyloid. **Right: left column (optical field 10×) and right column (optical field 20×):** *H&E*, severe amyloid infiltration in the interstitium and vessels with degeneration of myocytes; *Congo Red and polarized light microscope*, Congo Red binding to amyloid fibrils showing the typical apple-green birefringence and revealing predominant interstitial infiltration; amyloid infiltration involves the interstitial space and vessel walls, including the sinoatrial nodal artery; *IHC for TTR and FLCs*, positivity of transthyretin (left, 40×) and immunoglobulin light chain Kappa (right, 20×) in advanced cases of cardiac amyloidosis. H&E, Hematoxylin and Eosin; FLCs, Free Light Chains; IHC, Immunohistochemistry; TTR, Transthyretin.

For this study, the hearts were independently evaluated by two pathologists (R.B., D.R.) with specific expertise in cardiovascular (CV) diseases who were blinded to clinical information according to the standards and definitions proposed by the Committee of the Society for Cardiovascular Pathology and the Association for European Cardiovascular Pathology (8). After gross examination, samples were collected from five sites for each case: a short-axis slice at the mid-ventricular level was obtained from the inferior-posterior wall of the left ventricle (LV), the lateral wall of the right ventricle (RV) and the interventricular septum (IVS), and, in addition, samples were collected from the roof of the left atrium (LA) and the right atrium. No cardiac samples were taken from the interatrial septum. For each sample, the latero-lateral diameter was 2.5 cm, and the slice thickness was 0.4 cm. Histological sections were stained with haematoxylin and eosin (H&E) and Congo red and then carefully analyzed for the presence of amyloid infiltration in the myocardium and vessels. Samples undergoing the Congo red staining procedure were evaluated with a polarized light microscope (9) (Figure 1).

In amyloid-positive samples, the localization (“interstitial” or “vascular”) and distribution (“non-diffuse” or “diffuse”) of amyloid fibrils were evaluated. The extent of the amyloid deposition was assessed semi-quantitatively on a visual scale as mild, moderate, or severe, corresponding to <25, 25–50, and >50% of the myocardial surface area, according to the cut-off values adopted in previous studies (6, 10). In detail, the area containing amyloid was visually defined as a percent of total tissue area of the histological section. The pattern of amyloid infiltration in each specimen was classified as: (a) “non-diffuse,” amyloid burden <50% of the histological section with macro-areas of confluent accumulation; or (b) “diffuse,” amyloid burden >50% of the histological section with widespread infiltration in the absence of any macro-area of amyloid accumulation (6, 10). For each patient, the predominant patterns of amyloid distribution and localization were determined. Finally, the presence and severity of coronary artery disease (CAD) was assessed using a semi-quantitative ordinal scale (0–3) reflecting no (0) to critical (3) atherosclerosis, as previously suggested (6, 10).

### Characterization of Amyloid Deposits and Extracardiac Organs

Immunohistochemistry with kappa and lambda light chains antibodies, anti-TTR antibodies, anti-apolipoprotein AI and anti-serum amyloid A antibodies was performed on the most representative sample for each patient to characterize the amyloid deposits (Figure 1). In detail, the following sources of antibodies were used: Amyloid A, 901-125-021318, Biocare Medical; Apolipoprotein AI, Polyclonal antibody, 14427-1-AP Proteintech; Anti-Prealbumin antibody, ab9015, Abcam; Kappa Probe 800-2843, 5278678001, VENTANA; and Cytoplasmic Lambda mRNA Probe, 800-2844, 05278686001, VENTANA. The bone marrow, the kidneys, the spleen and the liver were evaluated to detect amyloid depositions on a regular basis in all patients with a post-mortem diagnosis of AL-CA and, in selected cases, in those with ATTR-CA.

### Cardiovascular Characterization of the Study Population

Data of clinical evaluations, electrocardiograms, echocardiograms, and blood tests performed within 12 months from the patients' deaths were extracted from the hospital's electronical database. The presence of carpal tunnel (CT) syndrome and previous CT surgery was assessed for each patient. Electrocardiographic and echocardiographic measures were based on standard definitions (11). In detail, ECG findings suggestive for CA, such as conduction abnormalities, low QRS voltages (≤ 0.5 mV in all limb leads and/or ≤ 1.0 mV in all precordial leads) and discrepancies between QRS voltages and the degree of cardiac hypertrophy at echocardiography (ECG-echo discrepancy) were evaluated. In patients with evidence of increased LV wall thickness at echocardiography, ECG-echo discrepancy was confirmed in absence of Sokolow–Lyon or Cornell criteria for LVH or in presence of low QRS voltages at the surface ECG. Echocardiographic images were analyzed for this study and independently reviewed by two cardiologists (A.P., M.M.) blinded to the histological presence of CA to evaluate the presence of the following recognized CA red flags: (a) restrictive filling pattern (E-wave deceleration time <120 or ≤ 150 ms in the presence of E/A ≥ 2) (12), (b) “granular sparkling” appearance of the myocardium (13), (c) pericardial effusion of any entity (14), (d) interatrial septum thickness >0.5 cm measured in the subcostal or four-chamber view (13), and (e) thickening of the atrio-ventricular valves (leaflet thickness > 0.5 cm) (15).

### Statistical Analysis

The time period for patient inclusion and the necessary number of autopsies were evaluated at the beginning of the study in regard to the aim of the analysis, the experience of pathologists in the CV field (R.B., D.R.) and the number of cardiac samples evaluated. In detail, considering an estimated disease prevalence of 20–25% according to previous autopsy studies conducted on similar populations of elderly patients, the required sample size to estimate the frequency of diseases with a CI 90% and an accuracy of ±8% was of 54 patients. Data are expressed as median and interquartile range (IQR) (25°; 75°); numbers and percentages were used when appropriate. Differences between groups were evaluated using the Mann–Whitney *U*-test for continuous variables and the chi-squared (χ^2^) or Fisher's exact-test for dichotomous variables. A *p*-value < 0.05 was considered statistically significant. All statistical analyses were performed using the IBM SPSS Statistics 24.0 package (New York, NY) statistical software version 20.

## Results

**Figure 2:**
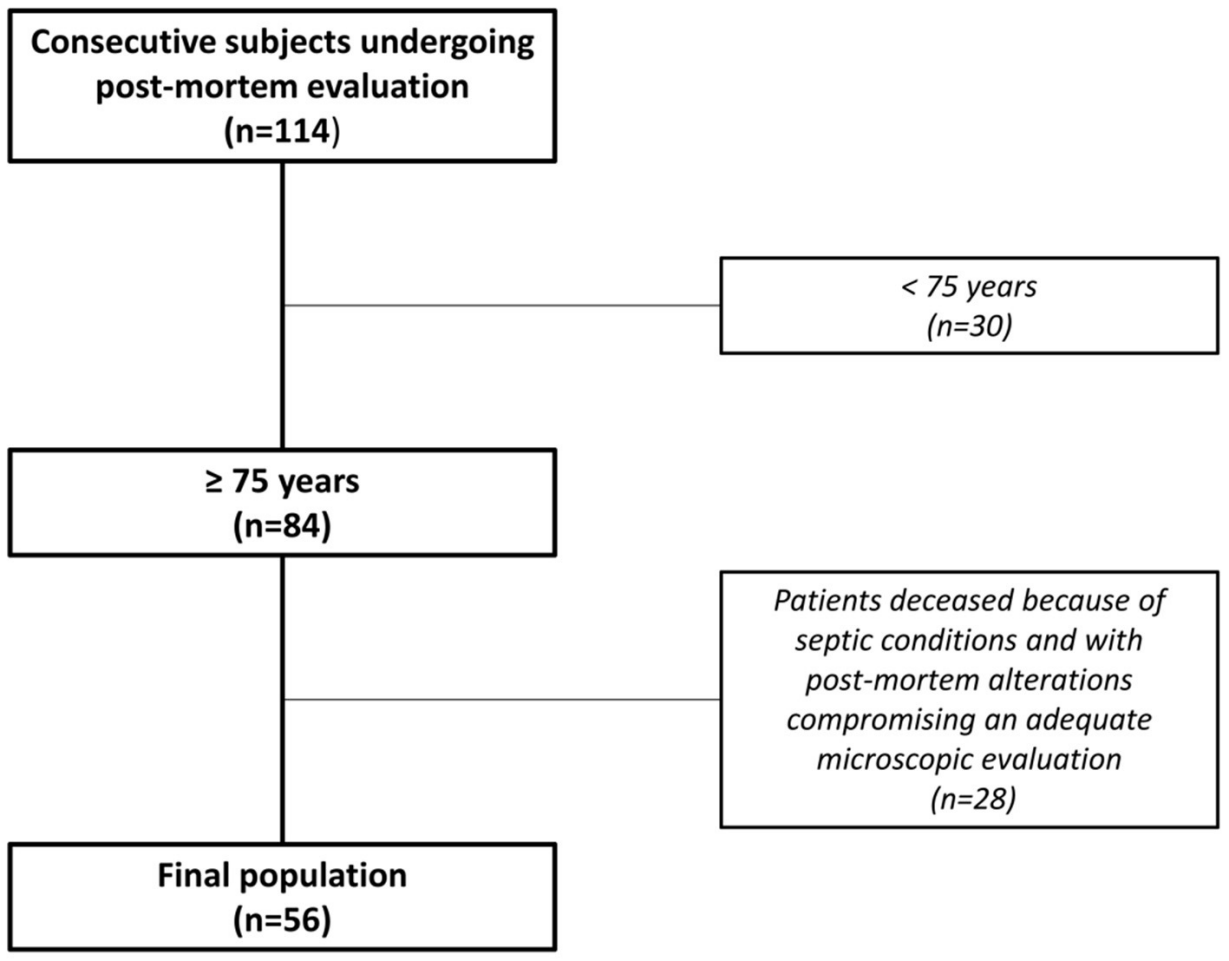
Study design. Flow-chart showing selection of the final population.

Of the 114 consecutive unselected subjects undergoing post-mortem evaluation over a 3-month period, 56 patients fulfilled the inclusion criteria and were included in the study population. Their median age was 86 years (IQR 82–90), 43% were men and 61% were diagnosed with HF (Figure 2). The baseline characteristics of the study population are shown in Table 1. At gross examination, the median heart weight was 480 gr (IQR 420–570), transverse heart diameter was 13 cm (IQR 11.5–14.0) and interatrial diameter was 6.5 cm (IQR 6.0–7.2).

**Table 1:** Characteristics of the study population based on histological evidence of CA.

**Parameters**	**Available data (*n*)**	**All (*n* = 56)**	**Non-CA (*n* = 32)**	**CA (*n* = 24)**	***P-*value**
Age at death, y	56	86 (82–90)	85 (81–89)	86 (84–91)	0.204
Male, %	56	43	38	50	0.350
ATTR, %	24	25	-	50	NA
AL, %	24	25	-	50	NA
BMI, Kg/mq	56	22 (20–25)	23 (20–26)	22 (20–24)	0.583
Hypertension, %	56	45	47	42	0.892
SBP, mmHg	56	120 (110–140)	120 (115–140)	125 (105–130)	0.297
Heart Failure, %	56	61	47	79	**0.014**
NYHA 3–4, %	34	14	6	25	**0.047**
Syncope, %	56	24	16	35	0.099
IHD, %	56	48	56	38	0.165
CKD (eGFR <60 ml/min)	56	73	81	63	0.117
**LABORATORY**					
Creatinin, mg/dL	54	1.03 (0.76–1.49)	0.94 (0.7–1.28)	1.15 (0.92–1.95)	*0.053*
eGFR, ml/min	54	60 (33–78)	66 (43–80)	53 (24–68)	**0.042**
Troponin T, ng/mL*	34	0.098 (0.026–1.388)	0.035 (0.017–0.11)	1.85 (1.30–3.96)	** <0.001**
BNP, pg/mL	31	394 (268–1,413)	302 (268–579)	935 (227–1,757)	0.147
Bilirubin, mg/dL	53	0.71 (0.51–1.10)	0.7 (0.47–1.15)	0.73 (0.52–1.08)	0.685
AST, U/L	54	25 (18–45)	25 (19–48)	25 (16–42)	0.581
ALT, U/L	54	16.5 (12–33)	16 (13–32)	17 (11–39)	0.793
**ELECTROCARDIOGRAPHY**					
AF, %	53	49	36	68	**0.019**
HR, bpm	53	78 (67–93)	78 (63–93)	80 (71–93)	0.545
LVH, %	53	35	33	36	0.820
RBBB, %	53	24	27	20	0.589
LBBB, %	53	16	10	25	0.156
Low QRS voltage, %	53	16	4	30	**0.009**
Discrepancy QRS-LVH, %	53	45	12	70	** <0.001**
**ECHOCARDIOGRAPHY**					
LVH (≥12 mm), %	37	66	39	100	** <0.001**
IVS, mm	37	12 (10–15)	11 (10–12)	15 (12–17)	** <0.001**
PWT, mm	37	11 (9–14)	11 (9–11)	12 (10–13)	**0.031**
RWT	37	0.46 (0.41–0.55)	0.41 (0.36–0.45)	0.49 (0.46–0.68)	**0.002**
LVEF, %	37	55 (49–60)	58 (53–63)	52 (38–59)	0.073
LVEF <50%, %	37	23	11	38	0.058
RV dysfunction, %	37	16	14	20	0.606
RFP, %	37	35	19	56	**0.020**
E/E′	37	13 (11–18)	12 (8–14)	17 (12–20)	*0.051*
LA diameter, mm	37	48 (41–52)	42 (36–50)	49 (45–54)	**0.019**
LA area, cmq	37	32 (27–36)	29 (24–35)	35 (32–36)	**0.021**
AVS,%	37	24	26	21	0.703
**AUTOPSY**					
Weight, gr	56	480 (420–570)	460 (409–518)	500 (420–600)	0.112
Transverse diameter, cm	56	13 (11.5–14)	12.8 (11.3–13.6)	13.5 (12–14)	0.097
IA diameter, cm	56	6.5 (6–7.2)	6.2 (5.5–7)	6.5 (6–7.3)	**0.048**
CAD 2–3^∧^, %	56	40	41	39	0.911

*At our Institution the normal value of serum troponin T <0.06 ng/mL.

*^∧^Semiquantitative ordinal scale (0–3) reflecting no (0) to critical (3) atherosclerosis.*

*AAL, Light Chain Amyloidosis; AF, Atrial Fibrillation; ATTR, Transthyretin Amyloidosis; AVS, Aortic Valve Stenosis; BAV I, First Degree Atrioventricular Block; BMI, Body Mass Index; CA, Cardiac amyloidosis; CAD, Coronary Artery Disease; CKD, Chronic Kidney Disease; DPB, Diastolic Blood Pressure; eGFR, estimated Glomerular Filtration Rate; IA, Interatrial; IHD, Ischemic Heart Disease; IVS, Interventricular Septum; LA, Left Atrial; LAFB, Left Anterior Fascicular Block; LBBB, Left Bundle Branch Block; LVEF, Left Ventricular Ejection Fraction; LVH, Left Ventricular Hypertrophy; PWT, Posterior Wall Thickness; RBBB, Right Bundle Branch Block; RFP, Restrictive Filling Pattern; RV, Right Ventricle; RWT, Relative Wall Thickness; SBP, Systolic Blood Pressure. Bold identifies significant p values (<0.05); Italics identifies p values near to the significance threshold*.

### Prevalence and Etiology of CA at Histology

**Figure 3:**
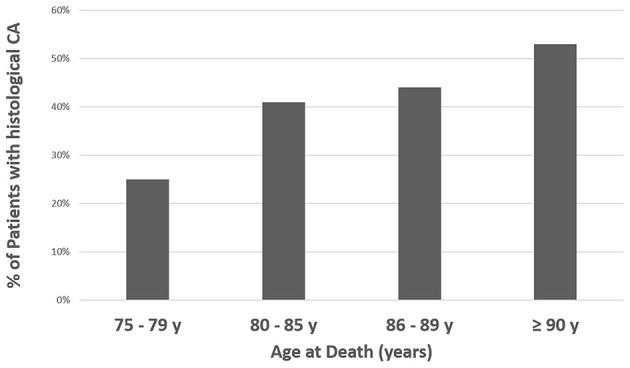
Prevalence of CA (any site) at autopsy according to age at death. %, percentage; CA, Cardiac Amyloidosis.

**Figure 4:**
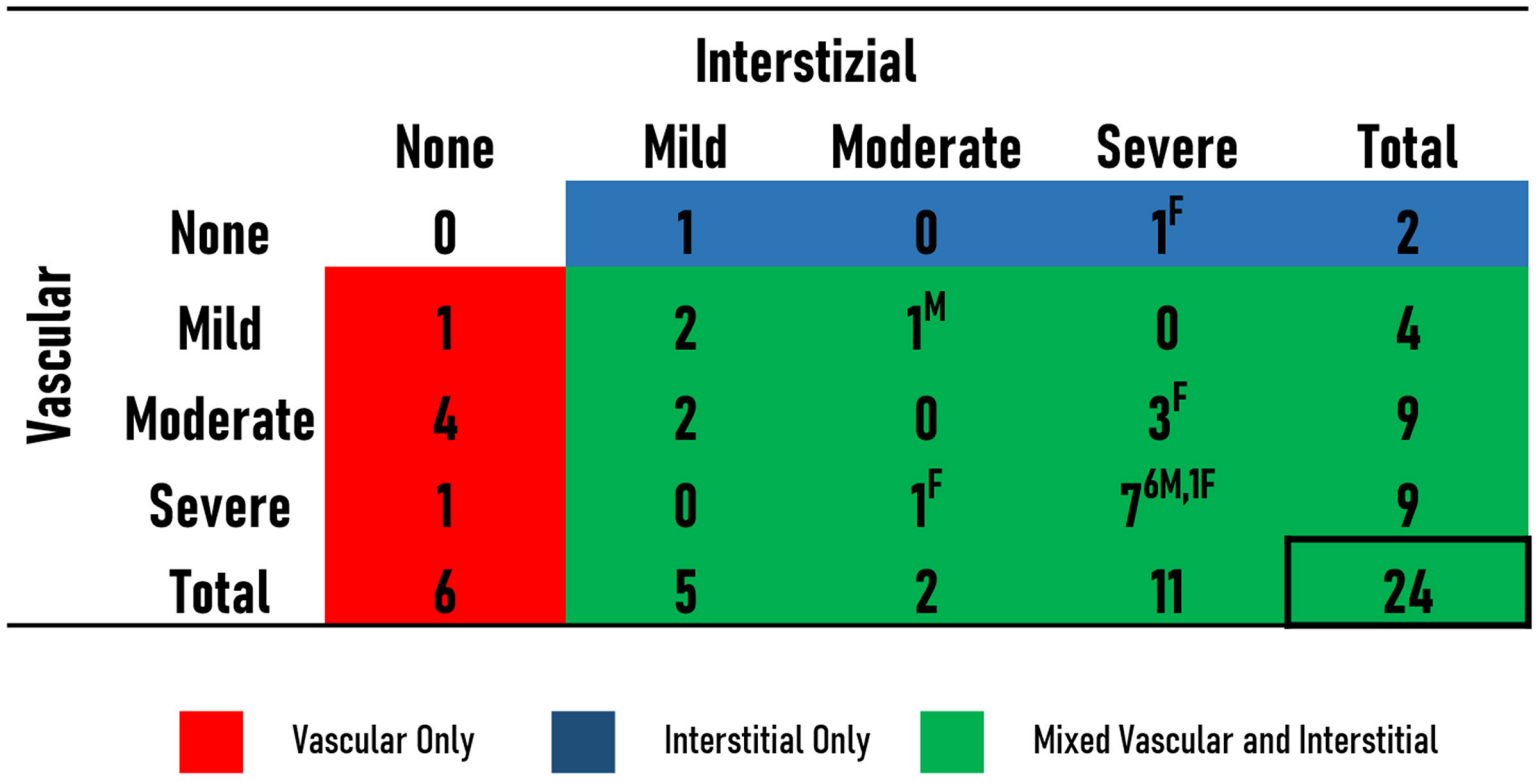
Localization and severity of amyloid deposition in the patients with CA. The number of patients with no (none), mild, moderate or severe interstitial and vascular amyloid is shown. Patients with only vascular deposition (red), only interstitial (blue), or mixed (green) deposition are illustrated. Sex [male (M) or female (F)] of patients with moderate and severe deposition is provided.

CA was found in 43% (*n* = 24/56 patients) of the autopsied hearts and was identified as the main cause of death in eight patients (14% of the study population; 33% of all patients with CA). In detail, these eight patients died due to advanced HF and/or cardiac arrest due to electromechanical dissociation and were referred to post-mortem evaluation by Internal Medicine and Geriatrics Departments. In the remaining cases, CA coexisted with other conditions contributing to death: myocardial ischemia at histological evaluation and advanced neoplasms (lung, skin, gut, and bone marrow). CA prevalence approached 25% in patients aged 75–79 years and increased with aging (Figure 3). At gross examination, compared to non-amyloid hearts, the amyloid hearts exhibited larger interatrial diameters [6.5 cm (6–7.3) vs. 6.2 cm (5.5–7), *p* = 0.048], while heart weights and transverse diameters were similar (*p* = 0.112 and *p* = 0.097; respectively) (Table 1). The atria were the most frequently infiltrated site (96%), followed by the LV (88%), the IVS (80%), and the RV (71%) (Table 2). Isolated atrial amyloidosis was found in a single case with ATTR-CA. Amyloid localized frequently at the vascular (95.5%, *n* = 22/24 hearts) and interstitial (85%, *n* = 18/24 hearts) levels (Figure 4). In detail, mild, moderate and severe amyloid accumulations were found in 58, 0, and 42% of the patients with CA, respectively.

**Table 2:** Prevalence, localization, and distribution of amyloid in heart chambers.

**Heart chamber**	**Presence of amyloid**	**Amyloid localization vascular vs. interstitial**	**Amyloid distribution non-diffuse vs. diffuse**
Atria	96% (23/24 pts)	91 vs. 78%	57 vs. 43%
LV	88% (21/24 pts)	95 vs. 76%	65 vs. 35%
IVS	80% (20/24 pts)	95 vs. 80%	57 vs. 43%
RV	71% (17/24 pts)	94 vs. 82%	71 vs. 29%

*IVS, Interventricular Septum; LV, Left Ventricle; pts, patients; RV, Right Ventricle*.

In terms of etiology, CA was equally related to AL and ATTR. Anti-apolipoprotein AI and anti-serum amyloid A antibodies were negative in all amyloid-positive samples. No difference between AL and ATTR was found in patients' median age at death, gross pathological evaluation, amyloid localization or distribution.

### Characteristics of CA and Non-CA Patients

Patient data, including clinical examination, electrocardiogram and echocardiogram, were available in 56 (100%), 53 (95%, 22 CA and 31 non-CA) and 37 (66%, 16 CA and 21 non-CA) patients, respectively, at a median time of 7 (IQR 3–10) months before autopsy. No patient with histological evidence of CA received a diagnosis or was suspected of the disease while alive. Patients with and without CA had similar median ages at death (*p* = 0.204) and gender distribution (*p* = 0.350). No patient had a previous history of CT syndrome or surgery. Compared to non-CA patients, those with histological evidence of CA were more frequently diagnosed with HF (79 vs. 47%, *p* = 0.014) with advanced NYHA functional class (III-IV class 25 vs. 6%, *p* = 0.047), higher serum troponin T values [1.85 (1.30–3.96) ng/mL vs. 0.035 (0.017–0.11) ng/mL, *p* < 0.001] and lower glomerular filtration rate [53 (24–68) ml/min vs. 66 (43–80) ml/min, *p* = 0.042]. They demonstrated greater rates of AF (68 vs. 36%, *p* = 0.019), low QRS voltages on surface ECG (30 vs. 4%, *p* = 0.009) and ECG-echo discrepancy (70 vs. 12%, *p* < 0.001). They showed increased rates of hypertrophic LV [IVS ≥ 12 mm in 100 vs. 39%, *p* < 0.001; IVS 15 mm (12–17) vs. 11 mm (10–12), *p* = 0.011; relative wall thickness (RWT) >0.42 in 100 vs. 50%, respectively, *p* = 0.003], enlarged LA diameter [49 mm (45–54) vs. 42 mm (36–50), *p* = 0.019] and restrictive LV filling pattern (56 vs. 19%, *p* = 0.020). CAD was found in all cardiac samples with a similar prevalence among CA and non-CA patients (*p* = 0.911). At univariate analysis, the presence of HF, AF, low QRS voltages and echo mass/QRS voltage discrepancy, cardiac hypertrophy, and restrictive filling pattern portended an increased risk of underlying CA (Table 3).

**Table 3:** Univariate analysis for the presence of amyloid deposition.

**Parameter**	**Odds ratio**	**95% CI**	***p-*value**
Age at death, per year	1.073	0.971–1.186	0.165
Men	1.667	0.570–4.876	0.351
Syncope	2.800	0.798–10.393	0.106
SBP, per mmHg	0.986	0.954–1.018	0.376
**Heart failure**	**4.307**	**1.290–14.373**	**0.018**
eGFR, per ml/min	0.995	0.972–1.018	0.655
HFpEF	1.636	0.498–5.379	0.417
NYHA 3–4	3.231	0.551–18.956	0.194
**Atrial fibrillation**	**3.896**	**1.221–12.431**	**0.022**
**ECG/echo discrepancy**	**17.143**	**3.063–95.938**	**0.001**
**Low QRS voltage**	**11.813**	**1.329–104.982**	**0.027**
LVEF <40%	1.583	0.274–9.166	0.085
LVEF, per %	0.944	0.885–1.006	0.074
RV dysfunction	1.583	0.274–9.166	0.608
**IVS thickness, per mm**	**1.915**	**1.219–3.009**	**0.005**
**Restrictive filling pattern**	**5.464**	**1.256–23.774**	**0.024**
Heart weight, per gr	1.005	1.000–1.010	0.054

*CI, Confidence Interval; eGFR, estimated Glomerular Filtration Rate; HFpEF, Heart Failure with Preserved Ejection Fraction; IVS, Interventricular Thickness; LVEF, Left Ventricular Ejection Fraction; NYHA, New York Heart Association; RV, Right Ventricular. Bold identifies significant p values (<0.05)*.

### Specific Characterization: AL vs. ATTR, Echocardiographic Red-Flags, RV Involvement, and Extracardiac Amyloid Deposition

Compared to ATTR patients, AL patients had lower values of systolic [110 (103–126) mmHg vs. 130 (115–140) mmHg, *p* = 0.033] and diastolic pressure [60 (60–70) mmHg vs. 80 (70–80) mmHg, *p* = 0.021] and higher values of RWT [0.68 (51–83) vs. 0.44 (0.36–0.44), *p* = 0.007]. There were no other differences between AL and ATTR patients.

In the 16 patients with histologically proven CA and reviewable echocardiographic images, the prevalence of echocardiographic CA red flags was as follows: 56% LV restrictive filling pattern, 56% thickened IAS, 38% thickened AV valves, 38% granular sparkling and 25% pericardial effusion. At least 3 echocardiographic CA red flags were present at the last echocardiography for 31% of the patients. No gender difference was found in their prevalence. Although showing a similar overall frequency of atrial amyloidosis, patients with thickened IAS at echocardiography had a higher frequency of diffuse amyloid infiltration in the atria compared to those without thickened IAS (67 vs. 14%, *p* = 0.036) (Figure 1). There were no morphological differences between patients with and without thickened IAS. Among the CA patients, compared to those without RV amyloidosis, patients with RV amyloid infiltration were predominantly women (86 vs. 35%, *p* = 0.025), were older at death [88 (85–93) vs. 84 (81–86) years; *p* = 0.048], had a more advanced NYHA functional class (3–4 classes 43 vs. 0%, *p* = 0.049) and showed increased rates of AF (69 vs. 41%, *p* = 0.050). At the histological level, most of the cases with RV amyloid infiltration had systematic LV amyloidosis and IVS amyloidosis compared to those without RV amyloidosis (100 vs. 0%, *p* = 0.004 and 94 vs. 54%, *p* = 0.027, respectively). Although showing similar rates of interstitial and vascular amyloid infiltration (*p* = 0.195 and *p* = 0.489, respectively), hearts with RV amyloidosis had a higher frequency of severe interstitial (63 vs. 0%, *p* = 0.005) and vascular (53 vs. 0%, *p* = 0.015) amyloid localizations, as well as diffuse amyloid distribution (60 vs. 0%, *p* = 0.008) compared to those without RV amyloidosis.

The evaluation of extracardiac organs revealed amyloid deposits in all AL-CA patients. Of note, some had been diagnosed with lymphoma (three patients), chronic leukemia (two patients) and MGUS (five patients) while alive, despite no one receiving a diagnosis or being deemed at suspicion of CA. Conversely, when extracardiac organs were evaluated in the ATTR-CA patients, no trace of amyloid was detected.

## Discussion

### Main Findings

To the best of our knowledge, this is the largest cohort of unselected patients ≥75 years old having been autopsied that has been systematically and extensively investigated at the histological level for the presence of CA and that has been characterized in terms of cardiological profile and correlation with histological data. The main findings of our study are that (a) CA can be found in up to 43% (24/56) of hearts from unselected subjects ≥75 years old who underwent autopsies, (b) AL-CA was as frequent as ATTR-CA, (c) CA patients more frequently had a history of HF with advanced NYHA functional class, AF with more dilated atria and LV concentric hypertrophy with thicker walls, (d) patients with RV amyloidosis had more severe cardiological profiles and more heavily infiltrated hearts at histology, (e) no patient received a diagnosis of CA, even though ECG-echo discrepancy and at least 3 red flags of CA were present in 70% and in more than 30% of patients with histologically proven CA, respectively.

These findings suggest that many individuals with CA, both AL and ATTR, are currently undiagnosed and that awareness of the disease's specific red flags is low in the medical community since no patient received a diagnosis of CA while alive, despite having suggestive ECG and echocardiographic features. Our results are even more relevant considering that the study was conducted over a 3-month period. Therefore, elderly patients with HF, AF and cardiac hypertrophy at echocardiography represent a population at increased likelihood of underlying CA and should be accurately evaluated for the presence of specific red flags (16). Furthermore, ATTR showed a predilection for the heart compared to extracardiac organs, as previously reported (17, 18). Although genetic testing for ATTR mutations was not performed, the advanced age of the population and the isolated cardiac involvement pointed toward a likely diagnosis of wild-type ATTR.

The real epidemiology of CA is currently unknown, particularly regarding ATTR. Historical post-mortem investigations on heterogeneous cohorts have reported CA predominantly in elderly patients (22–25% of subjects >80 years old) (4, 19–22). Recent autopsy studies in patients >75 years old with an antemortem diagnosis of HFpEF without clinically apparent amyloid found CA in 14–32% of cases (mostly ATTR) (6, 23, 24). The present study is in line with these reports, showing CA in ≈25% of patients <80 years old (Figure 3). Furthermore, our results suggest a higher prevalence of CA in patients ≥75 years old as long as an accurate characterization of multiple cardiac samples is performed by experienced cardiac pathologists, including specific staining procedures and immunohistochemistry. A number of reasons might explain the higher CA prevalence found in the present analysis compared to previous autopsy studies: different enrolled cohorts, heterogeneous sites of collection and number of cardiac samples, and number of CV pathologists analyzing the specimens (for example, only one sample from the LV inferior wall was analyzed by a single pathologist in some studies) (4, 6, 24). Unlike other investigations, we included an unselected population and systematically collected samples from five different cardiac sites (one for each cardiac chamber + IVS), even in patients without known cardiac disease while living (Figure 1). Surprisingly, AL-CA was found in a significant quota of patients ≥75 years old and thus should be considered in elderly patients, along with ATTR. These data are in line with previous investigations identifying AL as the cause of CA in up to 41% of elderly patients (median age 79 years), amenable to specific treatments (i.e., chemotherapy) (25).

In recent years, the implementation of bortezomib in chemotherapy regimens for AL (26) and the recent availability of tafamidis for patients with ATTR-CA have revolutionized the therapeutic approach to CA. Of note, in the ATTR-ACT trial, the positive results of tafamidis (30% reduction in all-cause mortality and CV-related hospitalizations) were demonstrated in a population of elderly patients (median age 75 ± 7 years) (27). Therefore, reaching a definite diagnosis and starting a disease-modifying drug treatment can translate into a concrete survival benefit, even in this population. Nevertheless, the selection of candidates—in particular, patients with early stages of the disease, mainly diagnosed non-invasively by scintigraphy—who might benefit most from treatment initiation is essential (28).

### Need of Systematic Evaluation of Red Flags of CA for an Early Diagnosis

Aging is associated with the failure of protein homeostasis and increasing rates of protein misfolding (29, 30), thus making it hard to establish whether the presence of amyloid in the hearts of elderly patients is an innocent bystander or a causative agent. In our study, the vast majority of CA patients (79%) had signs and symptoms of HF and AF and echocardiographic evidence of significant cardiac pseudo-hypertrophy due to amyloid infiltration, thus reflecting a true cardiac disease. The critical interpretation of ECG and echocardiographic findings of patients with post-mortem evidence of CA would have detected the discrepancy between QRS voltages and the degree of ventricular thickness and the specific red flags of infiltrative disease, thus raising the suspicion of CA while they were alive. Therefore, even in the absence of clinically overt HF, the presence of red flags in the work-up of unexplained cardiac hypertrophy should prompt further testing to identify concomitant diseases such as CA. This is far more relevant considering that defining the amount of “significant” amyloid burden is challenging since even low amyloid amounts might have detrimental consequences in vulnerable areas such as the atrio-ventricular node.

Patients in this analysis had severe CA; hence, making the correct diagnosis at their last evaluation before death would not have changed their natural history. However, these findings reflect the ominous natural history of untreated CA patients diagnosed in advanced stages of the disease (31). Therefore, there is an urgent need to promote awareness of the disease among physicians of several specialties since many patients with post-mortem evidence of CA could have been considered at suspicion of CA or even diagnosed non-invasively during their lives.

### The RV in CA: Passive Actor or Prudent Sentinel?

Our results suggest that RV infiltration could be a valuable marker of global amyloid burden in the heart and might occur in advanced stages of the disease. In this study, histological RV amyloidosis was found in >70% of CA patients and seemed to identify more heavily infiltrated hearts at both the clinical and histological levels. Under the microscope, all hearts with evidence of RV amyloidosis also showed LV amyloid infiltration (biventricular amyloidosis) as well as more severe interstitial and vascular amyloid deposits, mostly with a diffuse pattern of distribution, consistent with heavily infiltrated hearts. Hence, the RV of CA patients should be carefully evaluated by echocardiography as it might provide prognostic information (32).

### Limitations

The study is monocentric and retrospective. Although we recognize the presence of a selection bias in the study population, its magnitude is limited since our Institute of Pathological Anatomy and Histology has the longest tradition of post-mortem examinations in Italy, and ~80% of inpatients deceased at our Institution undergo autopsy each year. Although limited in number, this sample represents the largest modern cohort of patients accurately and extensively characterized at the histopathological and cardiological levels. Histological examinations were performed by pathologists with a solid experience in the CV field who were specifically asked to search for amyloid infiltration in multiple cardiac samples undergoing dedicated staining procedures. This might explain the higher prevalence of CA detected in this study. The presence of amyloid deposition at the level of the atrial appendage, cardiac valves and the conduction system was not investigated, being outside the scope of the present study. Due to post-mortem diagnosis of CA, no patient underwent TTR sequencing while alive. However, based on epidemiological data in our geographical area (i.e., North-East of Italy), it is very likely that all patients included in this analysis and diagnosed with ATTR-CA had a wild-type TTR. Alpha-actin staining might have provided additional information for amyloid vascular localization, but it is not routinely performed at our Center. Defining the amount of “significant” amyloid burden with specific thresholds is difficult, and there are no defined cut-off values for mild, moderate or severe amyloid infiltration. Therefore, we adopted those used in the few previous studies (6, 10). Mass spectrometry is not available at our Center, and characterization of the amyloid deposits was performed by immunohistochemistry with specific antibodies. However, mass spectrometry is not essential in most cases to reach a diagnosis or to identify the etiology of CA. Although suffering from technical limitations (33), immunohistochemistry is the most diffuse method of analysis, while mass spectrometry suffers from limited availability (9). Finally, cardiological data were not available for all patients.

## Conclusion

CA can be found in 43% of autopsied hearts from unselected patients ≥75 years old, evenly distributed among ATTR and AL, as long as cardiac samples are collected from multiple sites. Most CA patients had a history of HF with advanced NYHA functional class and suggestive clinical and instrumental findings. Notably, recognition of red flags is still poor in the medical community since >30% of patients with histologically proven CA had echocardiograms highly suggestive of the disease at their last evaluations and could have been diagnosed while alive.

## Data Availability Statement

The data underlying this article cannot be shared publicly for the privacy of individuals that participated in the study. The data will be shared on reasonable request to the corresponding author.

## Ethics Statement

All patients signed an informed consent document to allow for the use of anonymized personal information for research purposes.

## Author Contributions

AP and MM contributed to the interpretation of data and to drafting the work. RB, DR, and GV contributed to the acquisition of data. AP, MM, LP, and GS contributed to the analysis and revision of the work. AP, RB, and GS contributed to the conception of the work. All authors contributed to the article and approved the submitted version.

## Conflict of Interest

The authors declare that the research was conducted in the absence of any commercial or financial relationships that could be construed as a potential conflict of interest.

## Publisher's Note

All claims expressed in this article are solely those of the authors and do not necessarily represent those of their affiliated organizations, or those of the publisher, the editors and the reviewers. Any product that may be evaluated in this article, or claim that may be made by its manufacturer, is not guaranteed or endorsed by the publisher.
